# Is Housing Quality Associated with Malaria Incidence among Young Children and Mosquito Vector Numbers? Evidence from Korogwe, Tanzania

**DOI:** 10.1371/journal.pone.0087358

**Published:** 2014-02-05

**Authors:** Jenny X. Liu, Teun Bousema, Brittany Zelman, Samwel Gesase, Ramadhan Hashim, Caroline Maxwell, Daniel Chandramohan, Roly Gosling

**Affiliations:** 1 Global Health Group, Global Health Sciences, University of California, San Francisco, San Francisco, California, United States of America; 2 Department of Immunology & Infection, London School of Hygiene and Tropical Medicine, London, United Kingdom; 3 Department of Medical Microbiology, Radboud University Nijmegen Medical Centre, Nijmegen, Netherlands; 4 Tanga Research Center, National Institute for Medical Research, Tanga, Tanzania; 5 Mwanza Interventions Trial Unit Center, National Institute for Medical Research, Mwanza, Tanzania; 6 Liverpool School of Tropical Medicine, Liverpool, United Kingdom; 7 Department of Epidemiology and Biostatistics, University of California, San Francisco, San Francisco, California, United States of America; Johns Hopkins University, United States of America

## Abstract

**Background:**

Several studies conducted in Northeast Tanzania have documented declines in malaria transmission even before interventions were scaled up. One explanation for these reductions may be the changes in socio-environmental conditions associated with economic development, and in particular improvements in housing construction.

**Objective:**

This analysis seeks to identify (1) risk factors for malaria incidence among young children and (2) household and environmental factors associated with mosquito vector numbers collected in the child’s sleeping area. Both analyses focus on housing construction quality as a key determinant.

**Methodology:**

For 435 children enrolled in a larger trial of intermittent preventive treatment for malaria in infants in the Korogwe District in Tanga, Northeastern Tanzania, detailed information on their dwelling characteristics were collected in the last year of the trial. Principal components analysis was used to construct an index of housing structure quality and converted to quintile units for regression analysis. Univariate and multivariate random effects negative binomial regressions were used to predict risk factors for child malaria incidence and the mean total number of indoor female *Anopheles gambiae* and *funestus* mosquitoes collected per household across three occasions.

**Findings:**

Building materials have substantially improved in Korogwe over time. Multivariate regressions showed that residing in rural areas (versus urban) increased malaria incidence rates by over three-fold and mean indoor female *A. gambiae* and *funestus* numbers by nearly two-fold. Compared to those residing in the lowest quality houses, children residing in the highest quality houses had one-third lower malaria incidence rates, even when wealth and rural residence were controlled for. Living in the highest quality houses reduced vector numbers while having cattle near the house significantly increased them.

**Conclusions:**

Results corroborate findings from other studies that show associations between malaria incidence and housing quality; associations were concentrated amongst the highest quality houses.

## Introduction

Recently, researchers have documented substantial declines in malaria incidence in eastern sub-Saharan Africa in areas that historically have had high malaria burdens. In coastal Kenya, pediatric malaria admissions have fallen steadily since 1999 and especially dramatically in Kilifi and Kwale [1]. Going further south along the coast, in northeast Tanzania, malaria transmission rates (assessed through seroconversion rates) among those born since 1998, have dropped [2] and the overall prevalence of malaria parasitaemia and the incidence of febrile malaria episodes have dropped by over 80% between 2003 and 2008 [3], [4]. The incidences of severe malaria and bacteremia have also rapidly fallen [5].

**Figure 1:**
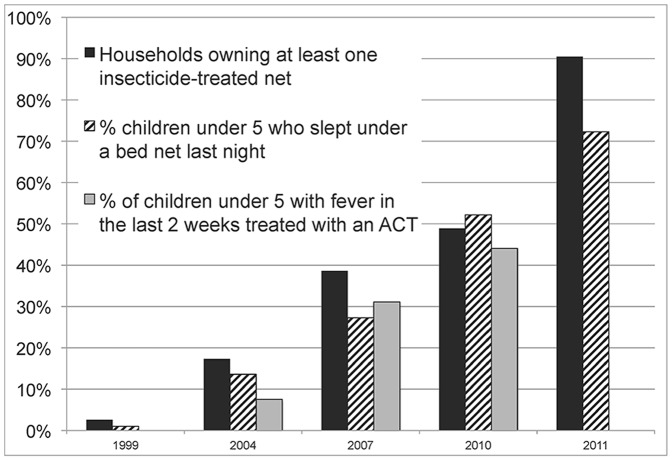
Selected malaria indicators from Tanzania Demographic and Health Surveys (1999, 2004, 2007, 2010, and 2011). Malaria intervention coverage rates have only recently increased. Greater household ownership of ITNs, ITN use among children under 5, and the proportion of children receiving artemisinin combination therapy (ACT) treatment in Tanzania started in 2004, surpassing 50% coverage only in 2010. Source: Tanzania Demographic and Health Surveys 1999, 2004, 2007, 2010, and 2011.

While some suggest that these improvements are attributable to increases in access to quality malaria diagnosis and treatment and preventive measures (e.g. insecticide-treated nets (ITNs), indoor residual spraying) in the region. However, intervention coverage rates have only recently increased: greater household ownership of ITNs, ITN use among children under 5, and the proportion of children receiving artemisinin combination therapy (ACT) treatment in Tanzania started in 2004, and only surpassed 50% coverage in 2010 (see Figure 1). ITN voucher programs for pregnant women and children began in late 2004 with only modest increases in household ownership (from 51% to 57% between 2004 and 2007) [6]. Effective treatment with ACTs was not available in government clinics until 2006 [7]. Thus, even though increasing intervention coverage may have facilitated the recent declines in malaria in the region, it is unlikely to be the only explanation for the longer-run trend.

Alternatively, the early decline in malaria may be related to the rapid changes in socio-environmental conditions associated with economic development in the area. As standards of living improve, individuals are able to spend more on lifestyle improvements, including housing structure improvements and health inputs (preventive and curative measures). A number of studies have documented individual and household risk factors for malaria that highlight the importance of housing structure. For example, malaria infection among children in the Ethiopian highlands was associated with having an earthen roof, animals sleeping in the house, open eaves, an attached kitchen, and having one sleeping room [8]. Elsewhere, living close to water reservoirs [9], [10], [11], in traditional huts [12], [13] or deteriorating housing, or ones with cracked, mud walls [14], mud roofs [15], [16], with unscreened windows and open eaves [16], [17] significantly increases infection risk. Many of these environment risk factors are closely linked to poverty, but have been shown to have independent effects above and beyond wealth status.

This study has two main objectives: (1) identify risk factors associated with malaria incidence among young children and (2) identify key household and environmental factors associated with the number of female *Anopheles gambiae sensu latu* (*A. gambiae*) and *Anopheles funestus* (*A. funestus*) mosquitoes (the main malaria vectors in the area) in the bedroom of study participants in an larger intervention study [18]. A particular focus is paid to the quality of the construction materials of the house as key determinants in both analyses.

## Materials and Methods

### Ethics

This study was approved by the ethics review boards of the National Institute for Medical Research of Tanzania and the London School of Hygiene and Tropical Medicine. Witnessed, written informed consent was obtained from the caretaker of enrolled study children.

### Study Area and Sample Selection

This study uses data from a larger, longitudinal trial study of intermittent preventive therapy in infants (IPTi) in the Tanga Region of northeastern Tanzania. The IPTi study area and trial methods have previously been described elsewhere [18], [19]. Briefly, the IPTi trial took place in the Korogwe and Same districts, an area of moderate and seasonal malaria transmission. Between 2005 and 2008, enrolled children (one per household) were followed from 2 to 24 months of age at pre-defined time points (3, 4, 9, 10, 18, and 24 months of age) throughout the study period and had 24-hour access to study physicians and quality-assured malaria diagnosis by blood slide. The primary outcome measure for the IPTi trial was incidence of all episodes of clinical malaria, which was defined as either a history of fever during the previous two days or an axillary temperature greater than 37.5°C plus parasitaemia of any density.

For this study, a subset of 435 children was randomly chosen from the 1240 children still enrolled in the IPTi trial in the last year of observation in the Korogwe District. The random sampling was stratified across three groups based on the cumulative number of malaria episodes over the course of the trial: 0 malaria episodes, 1–2 episodes, and ≥3 episodes. In the last year of the IPTi trial (2007–2008), a survey was conducted for this subset of children to collect individual and household characteristics, household infrastructure, geo-location, surrounding environmental features, and characteristics of the sleeping area of the index child enrolled in the IPTi study. In addition, a historical account of housing improvements was collected by asking caregivers for the type of roofs and walls that their housing unit was made of at the time of different life events: when they were first married, when they had their first child, when they had their last child, and at the time of survey.

To collect information about exposure to mosquitoes, an indoor rapid vector assessment exercise was conducted in 2008 for all households of the sample subset of 435 children. For a detailed explanation of mosquito catch data, see Bousema et al. [19]. Adult mosquitoes were sampled by CDC light traps (Model 512; John W. Hock Company, Gainesville, Florida, USA) using standard protocols [20]. Each household was sampled for 1–2 nights on three occasions: at the end of the wet season (May), and at the beginning (July) and end of the dry season (September). If households were sampled more than once during one season (i.e. wet, beginning dry or end dry), the average of these sampling time-points was taken to have one observation per season. Sampling was halted after September due to completion of the main IPTi study. Every sampling night, a median of 14 mosquito traps (IQR 10–16) were set in the bedroom of enrolled children. This resulted in a total of 499 traps at the end of the wet season, 506 at the beginning of the dry season and 449 at the end of the dry season. The order in which households were sampled was determined by randomisation in blocks, ensuring that each night sampling was done in households of all three malaria episode strata (0 malaria episodes, 1–2 episodes and ≥3 episodes) in an approximate ratio of 4∶1:1.

### Descriptive Analysis

Using data collected on house materials at the time of major life events, the historical trend in housing improvements for the chosen subset of households was constructed. The cumulative proportion of study households changing from thatched roofs to iron roofs and from dirt floors to concrete floors was calculated across urban and rural areas separately.

To assess the association between house quality and malaria incidence, the number of positive blood slides per child per year (standardized for varying time periods of observation) was plotted against an index of the quality of the house resided in at the time the household survey was conducted in the last year of the IPTi trial. Principal components analysis (using only the first component) was conducted using the set of variables collected for house and room construction materials to construct a house quality index. The component items are as follows: roof material, wall material, wall texture, presence of eaves, floor finishing, degree of screening on windows, and presence of a ceiling. This index was then converted to quintile categorical indicators for use in data analysis. Auxiliary analysis showed that higher order principal components yielded little additional explanatory power in regression analyses and thus were excluded, and that univariate and multivariate associations hold when using different numbers of categories for the house index. To assess the association between house quality and mosquito numbers, the median number of female *A. gambiae* and *funestus* mosquitoes (mean total number collected across three occasions in each household) are plotted against house quality quintiles (urban and rural separately). The median of mean vector numbers was also plotted against the presence of cattle near the house–one risk factor emerging from multivariate regression outputs.

### Regression Analysis

Two types of regression analyses were conducted. First, we estimated univariate and multivariate negative binomial regressions to assess individual and household risk factors for malaria incidence. The number of slide positives per child per year was predicted by the child’s age, mother’s education, household wealth, infrastructure conditions (i.e. electricity, piped water), and rural/urban location. Dummy variables for currently living in a house corresponding to housing indices were included (the lowest quality house type is the reference category). Historic measures of housing improvements were not used as too few households changed building materials during the years when the IPTi trial was conducted. Malaria prevention measures that would influence the child’s exposure, including use of repellent and insecticide-treated nets were controlled for, as was the arm of the IPTi study that the child was randomized to. Only statistically significant explanatory variables at the 5% level in univariate analysis were included in the multivariate model. To account for spatial autocorrelation across geographic areas, village-level random effects were included. Within villages, households were classified as being located in “urban” hubs or outlying “rural” areas as identified by study surveyors when conducting household visits.

Second, the mean total number of indoor female *A. gambiae* and *funestus* mosquitoes collected from each household from three occasions was predicted using a negative binomial regression. Sensitivity analyses showed that mean total female *A. gambiae* and *funestus* mosquito numbers per household yielded more precise estimates of standard errors than regressions predicting each occasion’s collected number separately. A histogram of the per household mean total number of female *A. gambiae* and *funestus* mosquitoes did not reveal an excessive number of zeros. Risk factors for mosquito numbers include indicators for house quality, the presence of cattle near the house, distance to the nearest water body, infrastructure conditions, and rural/urban location. Only statistically significant explanatory variables at the 5% level in univariate analysis were included in the multivariate model. Village-level random effects were again included in all regressions to account for spatial autocorrelation.

## Results

The 435 children in this subset experienced about 1.5 episodes of malaria on average, or 0.8 episodes per child per year when adjusted for observation time. However, malaria incidence was highly skewed as 61% of children did not experience a single episode of malaria. When averaged over the three occasions in each household, the median number of all mosquitoes caught was about 19 (mean = 43.5; 95% CI: 37.5–49.4). Most mosquitoes were female *Culex* (median = 11.7; mean = 37.0; 95% CI: 31.2–42.8). Only a small portion was female *A. gambiae* (median = 1.3; mean = 4.1; 95% CI: 3.0–5.3) and *A. funestus* (median = 0.3; mean = 1.8, 95% CI: 1.4–2.3). Table 1 summarizes these outcome variables.

**Table 1:** Child malaria incidence and mosquito numbers collected per household.

Variable	N	Median	Mean	CI
**Slide positivity rate per** **child per year**				
Total	435	0.0	0.81	0.68–0.64
Urban	179	0.0	0.15	0.08–0.22
Rural	256	0.6	1.27	1.07–1.47
**Mosquito counts averaged** **over three occasions**				
All mosquitoes	435	19.0	43.5	37.5–49.4
Female *Culex*	435	11.7	37.0	31.2–42.8
Female *Anopheles*	435	2.0	6.1	4.6–7.6
Female *Anopheles* *gambiae s.l.*+*funestus*	435	2.0	6.0	4.5–7.4
Female *Anopheles gambiae s.l.*	435	1.3	4.1	3.0–5.3
Female *Anopheles funestus*	435	0.3	1.8	1.4–2.3

**Figure 2:**
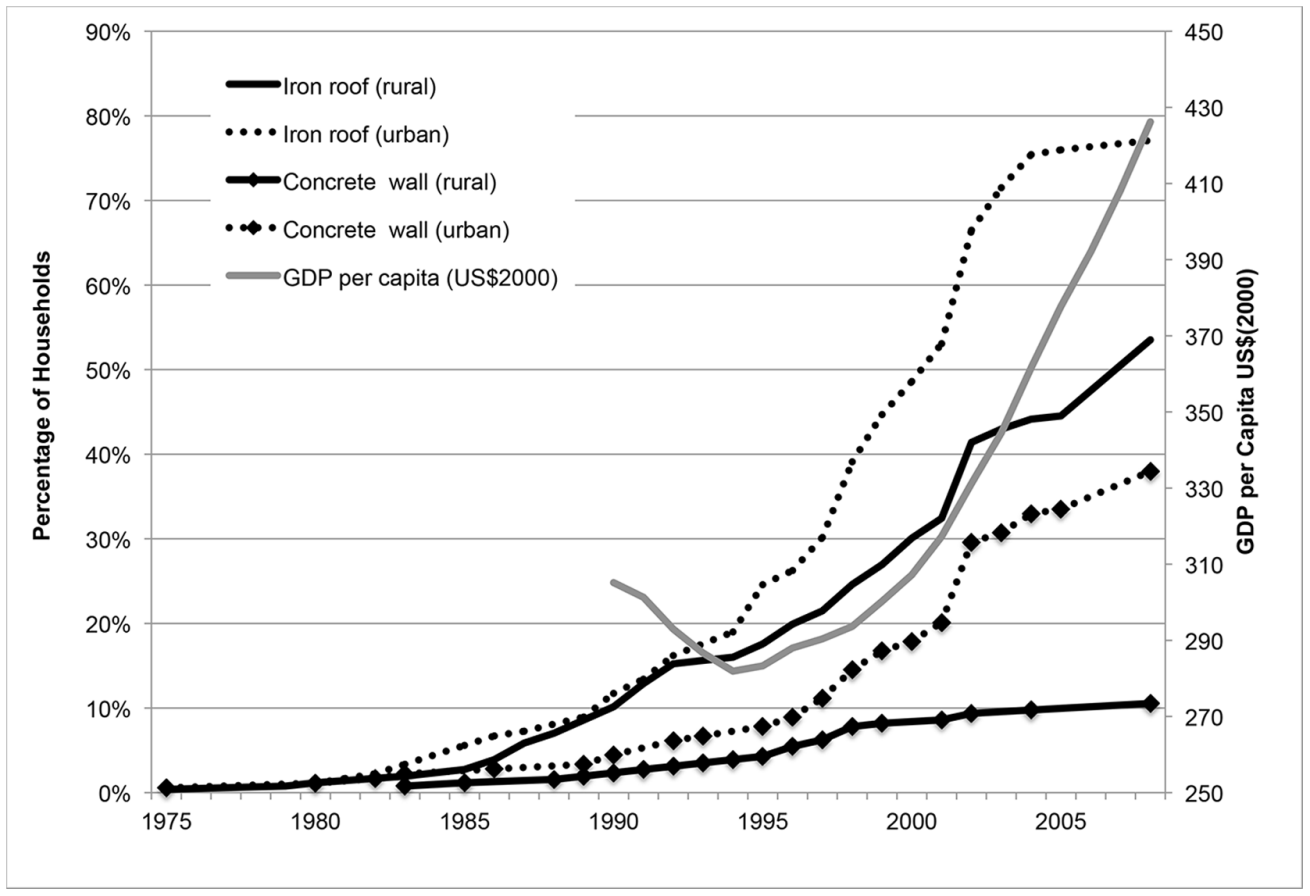
Proportion of households with concrete walls and iron roofs over time (1975–2008). A historical account of housing improvements was collected by asking respondents for the type of roofs and walls that their housing unit was made of at the time of different life events: when they were first married, when they had their first child, when they had their last child, and at the time of survey. These dates are used to calculate the cumulative density of houses that have attained iron roofs (instead of thatched roofs) and concrete walls (replacing mud walls) since 1975. Whereas the proportion of houses with iron roofs or concrete walls was nearly zero prior to 1985, by 2008, nearly 80% and 40% of houses have these materials, respectively. These trends correspond with increasing national gross domestic product per capita in the country. Notes: GDP per capita (US$2000) obtained from World Bank Indicators.

There has been a noticeable improvement in the quality of houses in the study area. Figure 2 displays the cumulative density of houses in Korogwe, Tanzania that have attained iron roofs (instead of thatched roofs) and concrete walls (replacing mud walls) since 1975. Whereas the proportion of houses with iron roofs or concrete walls was nearly zero prior to 1985, by 2008, nearly 80% and 40% of houses have these materials, respectively. While these proportions were lower in rural areas, building materials of houses nonetheless have also improved. These trends correspond with increasing national gross domestic product per capita, suggesting generally improving population living standards.

Table 2 lists the nine component items used to construct the house quality index, as well their percentage contributions across index quintiles. Houses with the lowest quality materials in quintile 1 all had thatched roofs, dirt floors, completely uncovered windows, and no ceilings; most of them had rough (n = 435; 100%) mud (n = 391; 90.0%) walls and open eaves (n = 367; 84.4%). In contrast, houses with the highest quality materials in quintile 5 nearly all had iron/tile roofs (n = 425; 97.6%), smooth (n = 425; 97.6%) concrete/brick (n = 361; 82.9%) walls, closed eaves (n = 382; 87.8%), finished floors (n  = 387; 89.0%), windows that are partially (n = 90; 20.7%) or fully (n = 318; 73.2%) screened, and ceilings (n = 244; 56.1%).

**Table 2:** Housing index component measures across quintiles.

	Housing index quintile				
Housing structure item	1	2	3	4	5
Iron/tile roof (vs. thatched/other)	0 (0%)	343 (78.9%)	333 (76.5%)	425 (97.8%)	425 (97.6%)
Concrete/brick wall (vs. mud/other)	44 (10%)	82 (18.9%)	140 (32.1%)	336 (77.2%)	361 (82.9%)
Smooth wall (vs. rough wall)	0 (0%)	19 (4.4%)	145 (33.3%)	355 (81.5%)	425 (97.6%)
Closed eaves (vs. open eaves)	68 (15.6%)	82 (18.9%)	75 (17.3%)	236 (54.3%)	382 (87.8%)
Finished floor (vs. unfinished floor)	0 (0%)	14 (3.3%)	129 (29.6%)	298 (68.5%)	387 (89%)
Windows: completely uncovered	435 (100%)	387 (88.9%)	226 (51.9%)	260 (59.8%)	27 (6.1%)
Windows: partially screened	0 (0%)	48 (11.1%)	129 (29.6%)	118 (27.2%)	90 (20.7%)
Windows: fully screened	0 (0%)	0 (0%)	80 (18.5%)	57 (13%)	318 (73.2%)
Has ceiling (vs. no ceiling)	0 (0%)	0 (0%)	11 (2.5%)	14 (3.3%)	244 (56.1%)

**Figure 3:**
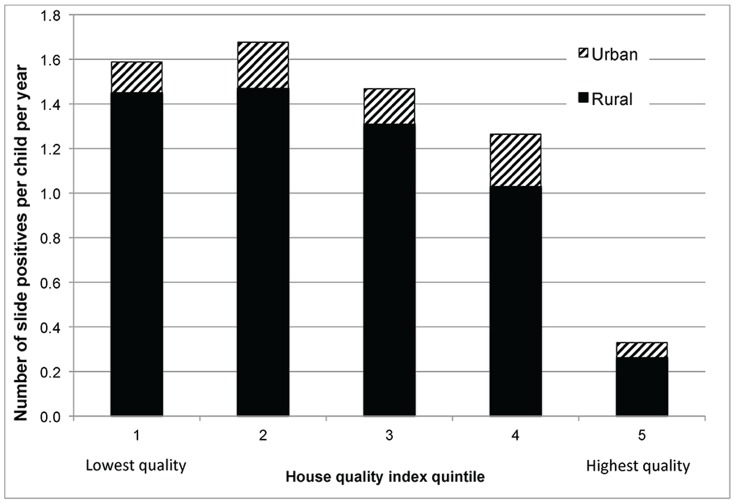
Slide positivity rate by housing index quintile. The incidence of malaria decreases as the housing quality index increases (Figure 3). In particular, incidence among children residing in the lowest three quintiles was over two to three times higher than among those residing in the highest quality quintile, and this was consistent across rural and urban areas.

The incidence of malaria (without regression controls) decreased as the housing quality index increased (Figure 3). In particular, incidence among children residing in the lowest three quintiles was over two to three times higher than among those residing in the highest quality quintile, and this was consistent across rural and urban areas.

**Figure 4:**
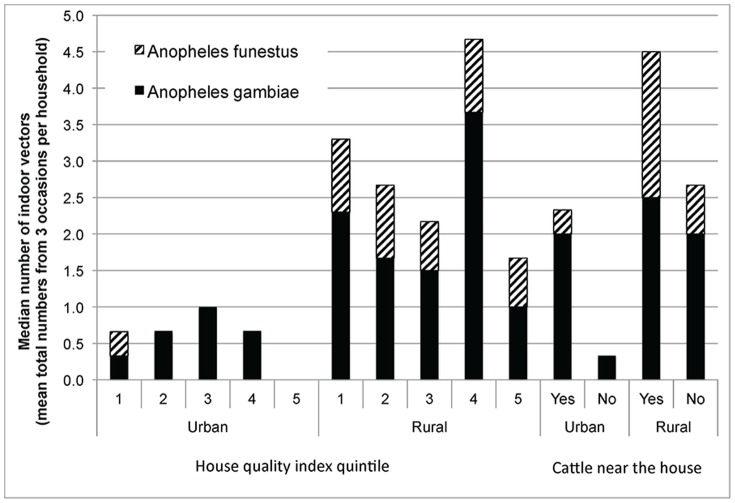
Total number of indoor mosquitoes vectors collected per household (mean of three occasions) by selected house quality indicators. There does not appear to be a consistent or gradient relationship between house quality and the mean mosquito numbers across urban and rural areas. In urban areas, the highest quality houses in quintile 5 had a median of zero vectors, and vectors were mainly comprised only of female *A gambiae*. In contrast, in rural areas, vector numbers were a mix of *A. gambiae* and *funestus*, and generally decreased as house quality increased, except for houses in quintile 4. More vectors were collected in houses with cattle nearby.

The median total number of vectors caught per household (mean of three occasions for each household) by housing index quintile is plotted in Figure 4. There does not appear to be a consistent or gradient relationship between house quality and mean mosquito numbers across urban and rural areas. In urban areas, the highest quality houses in quintile 5 had a median of zero vectors, and vectors were mainly comprised only of female *A gambiae*. In contrast, in rural areas, vector numbers were a mix of *A. gambiae* and *funestus*, and generally decreased as house quality increased, except for houses in quintile 4. More vectors were collected in houses with cattle nearby.

Regression results predicting child malaria incidence are shown in Table 3. In univariate analyses, children born in 2006, residing in households of higher wealth quintiles and houses in higher quality quintiles, who used bed nets, had access to piped water and electricity, and were located in urban hubs had significantly lower malaria incidence rates. In multivariate analyses, children living in rural areas had a 3.5-fold elevated malaria incidence rate (IRR = 3.577; 95% CI: 2.048–6.247). Compared to those living in the highest quality housing structures, and after adjustment for socio-economic status and other factors, children living in the highest quality houses (quintile 5) had a significantly lower malaria incidence rate (IRR = 0.324; 95% CI 0.148–0.708). Using a bed net significantly reduced the malaria incidence rate by about half (IRR = 0.446; 95% CI 0.329–0.604) after controlling for other confounders.

**Table 3:** Risk factors for malaria incidence among children.

		Univariate			Multivariate^2^		
Variables	N	IRR^1^	95% CI	P-val	IRR^1^	95% CI	P-val
**Child age**							
Age 2 or less (reference)	114	1.000			1.000		
Age 3	298	1.357*	0.958–1.921	0.086	1.087	0.774–1.526	0.629
Age 4	23	1.315	0.672–2.571	0.424	0.725	0.375–1.401	0.338
**Mother’s education**							
No schooling	44	1.144	0.743–1.763	0.541			
Primary or more (reference)	391	1.000					
**Wealth index**							
Poorest	103	1.756***	1.210–2.549	0.003	1.316	0.915–1.891	0.139
Poorer	81	1.498**	1.001–2.241	0.049	1.292	0.876–1.905	0.196
Middle (reference)	97	1.000			1.000		
Richer	67	0.954	0.570–1.594	0.856	1.090	0.667–1.782	0.732
Richest	87	0.448**	0.220–0.915	0.028	1.059	0.533–2.103	0.870
**Housing index**							
Quintile 1	90	1.000			1.000		
Quintile 2	90	0.856	0.604–1.214	0.383	1.015	0.729–1.412	0.932
Quintile 3	81	0.610**	0.397–0.938	0.025	0.844	0.560–1.273	0.419
Quintile 4	92	0.585**	0.387–0.884	0.011	0.858	0.578–1.274	0.448
Quintile 5	82	0.167***	0.0780–0.358	0.000	0.324***	0.148–0.708	0.005
**Regular repellent use**							
Less than once/week (reference)	375	1.000			1.000		
More than once/week	60	0.737	0.485–1.120	0.153	0.446***	0.329–0.604	0.000
**Bed net use last night**							
Yes	215	0.439***	0.327–0.589	0.000	0.446***	0.329–0.604	0.000
No (reference)	220	1.000			1.000		
**Water source**							
Piped water	60	0.365**	0.165–0.810	0.013	0.968	0.411–2.277	0.940
Other source (reference)	375	1.000			1.000		
**Electricity**							
Has electricity	84	0.358***	0.181–0.710	0.003	0.574	0.273–1.207	0.144
No electricity (reference)	351	1.000			1.000		
**Location type**							
Urban (reference)	179	1.000			1.000		
Rural	256	4.934***	2.865–8.498	0.000	3.577***	2.048–6.247	0.000
Observations		435			435		
Villages		15			15		

1 Incidence rate ratios (RRRs) estimated by random effects negative binomial regression specifying exposure time per child.

2 Regression also controls for the trial arm that the participant was randomized to: mefloquine, sulfadoxine-pyrimethamine, CD, control.

*** p<0.01, **p<0.05, *p<0.1.

Table 4 summarize the regression results for the mean total number of indoor female *A. gambiae* and *funestus* mosquitoes collected per house across three occasions. In univariate analyses, houses in the highest quality quintile, without cattle nearby, with piped water and electricity, and that are located in urban areas all had significantly lower mean vector numbers. In multivariate analysis, rural location (IRR = 1.713; 95% CI: 1.282–2.90) and having cattle near the house (IRR = 1.319; 95% CI: 1.056–1.647) significantly predicted higher mean *A. gambiae* and *funestus* numbers. A house in quality quintile 5 was significantly associated with lower vector numbers (IRR = 0.571; 95% CI: 0.373–0.874).

**Table 4:** Risk factors for the mean total number of indoor vectors per household collected over three occasions.

		Univariate			Multivariate		
Variables	N	IRR^1^	95% CI	P-val	IRR^1^	95% CI	P-val
**House quality index**							
Quintile 1	90	1.000			1.000		
Quintile 2	90	0.852	0.647–1.123	0.256	0.940	0.717–1.233	0.655
Quintile 3	81	0.769*	0.573–1.033	0.081	0.872	0.650–1.169	0.359
Quintile 4	92	0.804	0.603–1.071	0.136	0.983	0.740–1.307	0.907
Quintile 5	82	0.334***	0.228–0.489	0.000	0.571***	0.373–0.874	0.010
**Cattle near house**							
Yes	83	1.551***	1.239–1.943	0.000	1.319**	1.056–1.647	0.015
No	352	1.000			1.000		
**Distance to nearest water (meters)**	435	1.000	1.000–1.001	0.392			
**Water source**							
Piped water	60	0.401***	0.270–0.593	0.000	0.727	0.455–1.163	0.184
Other source (reference)	375	1.000			1.000		
**Electricity**							
Has electricity	84	0.459***	0.331–0.639	0.000	0.828	0.554–1.237	0.356
No electricity (reference)	351	1.000			1.000		
**Location type**							
Urban (reference)	179	1.000			1.000		
Rural	256	2.334***	1.787–3.048	0.000	1.713***	1.282–2.290	0.000
Observations		435			435		
Number of villages		15			15		

1 Incidence rate ratios (IRRs) estimated by random effects negative binomial regression.

Dependent variable is the mean total number of *Anopheles gambiae* and *funestus* caught per household over three occasions.

*** p<0.01, **p<0.05, *p<0.1.

## Discussion

This analysis of risk factors for malaria among young children suggests that there may be some role of urbanization and housing materials in reducing risk, even when controlling for socioeconomics differences and preventive behaviors. The incidence of malaria among urban children was nearly one-fourth of those living in rural areas. This may be related to the number of mosquito vectors found indoors. The main urban centre of Korogwe is a small rural town with a population of between 40,000–45,000. In this study, we classified the centre of each village as an “urban” hub with surrounding rural areas. Even when including these “urban” hubs in an overall very rural setting, indoor female *A. gambiae* and *funestus* catches were found to be significantly lower in urban hubs. This finding could represent “urbanization” at the village level where the density of housing and the pollution of breeding sites preferred by *A. gambiae* and *funestus* mosquitoes reduces malaria transmission. In The Gambia, significant reductions in vectors were also found among houses located within the town center compared to outlying areas [17].

The analysis also corroborates findings from other studies that show associations between malaria risk and poor housing materials [8], [11]–[12], [14]–[15], [21]. Housing materials have improved over time among households in the study area (i.e. Korogwe), especially in the last 20 years. Compared to those who live in houses that are in the lower quality four-fifths of structures, living in the highest quality house type significantly reduced malaria incidence and vector numbers. Additional analyses of the larger population of IPTi children also found significant associations between vector density and malaria hotspots in the study area [19]. Additional factors, such as having cattle near the house, may attract mosquitoes, but neither *A. gambiae* nor *A. funestus* is characterized by marked zoophilic behavior.

Entomological surveys in nearby villages in Tanga, Tanzania show a large decline in mosquitoes overall between 1998 and 2009 caught in indoor light traps [22]. Reductions in malaria transmission may also be related to general improvements in socioeconomic status that enable greater access to healthcare and personal protection measures, the product of which may be reflected in better housing structures. Proximity to health care was noted in the larger IPTi study area to be protective against malaria incidence [18]. Increasing access to antimalarial drugs that results in the frequent overtreatment approximating a massive chemoprophylaxis of the population may also decrease malaria transmission [23].

These results should be interpreted in light of additional caveats. Although analyses suggest that house quality was related to both malaria incidence and vector numbers, this relationship was only significant for the highest quality types. Even when examining multivariate regressions using different numbers of housing index categories, there were not significant differences between the majority of houses that were in lower quality tiers. This suggests that other risk factors not included in this analyses may better explain malaria incidence or vector numbers among these households and should be further explored in future analyses.

Could house improvements be an intervention to reduce malaria transmission? The protective effects of housing structure improvements such as house screening, closing of eaves, and ceiling installation have been documented since the 19th century [24]. Installation of ceiling netting has been shown to be cost-effective compared with provision of bed-nets, and reduced transmission by 80% in study areas [25], [26]. Other interventions that have screened doors and windows and closed eaves and wall holes have significantly reduced vector density [27], [28] and have been shown to be fairly inexpensive [28]. Entry point screens also reduce vectors for other infectious diseases [29], conferring larger benefits for household health beyond malaria [16].

In low transmission settings where countries are moving towards elimination and malaria becomes highly clustered [30] it may be cost-effective to make house improvements on the few poor housing structures in a malaria hotspot and render the site non-receptive to malaria [31]. Even though the costs of such housing structure interventions have higher up-front costs especially in areas of moderate and high transmission, they may be more cost-effective over time. Permanent change eliminates the need to continuously distribute LLINs, conduct IRS periodically, or demand consistent use of ITNs, which is particularly difficult to sustain when the perceived risk of malaria decreases [32]. However, more research is needed to ascertain which building improvements are the most effective.

Surveying household conditions could aid in defining and identifying hotspots and “hotpops,” or geographic areas or sub-populations where malaria risk is concentrated and where interventions should be targeted [33]. Improving these features may reduce mosquito exposure and malaria risk in these households, and more broadly appears to be related to gradual economic development. The effect of increasing wealth is likely to also facilitate increased use of protective measures, better health seeking behavior, as well as reduced exposure to other infectious diseases.

## References

[1] Okiro EA, Hay SI, Gikandi PW, Sharif SK, Noor AM, et al (2007) The decline in paediatric malaria admissions on the coast of Kenya. Malar J 6: 151. 18005422 10.1186/1475-2875-6-151 PMC2194691

[2] Stewart L, Gosling R, Griffin J, Gesase S, Campo J, et al (2009) Rapid assessment of malaria transmission using age-specific sero-conversion rates. PLoS ONE 4(6): e6083. 19562032 10.1371/journal.pone.0006083 PMC2698122

[3] Mmbando BP, Besergaard LS, Kitua AY, Lemnge MM, Theander TG, et al (2010) A progressive declining in the burden of malaria in north-eastern Tanzania. Malar J 9: 216. 20650014 10.1186/1475-2875-9-216 PMC2920289

[4] Winskill P, Rowland M, Mtove G, Malima RC, Kirby MJ (2011) Malaria risk factors in north-east Tanzania. Malar J 10: 98. 21507217 10.1186/1475-2875-10-98 PMC3094229

[5] Mtove G, Amos B, Nadjm B, Hendriksen IC, Dondorp AM, et al (2010) Decreasing incidence of severe malaria and community-acquired bacteraemia among hospitalized children in Muheza, north-eastern Tanzania, 2006–2010. Malar J 10: 320. 10.1186/1475-2875-10-320 PMC3219788 22029477

[6] Hanson K, Merchant T, Nathan R, Mponda H, Jones C, et al (2009) Household ownership and use of insecticide treated nets among target groups after implementation of a national voucher programme in the United Republic of Tanzania: plausibility study using three annual cross sectional household surveys. BMJ 339: b2434. 19574316 10.1136/bmj.b2434 PMC2714691

[7] Mbuyazi GM, Gonzalez-Bock MA (2005) Research influence on antimalarial drug policy change in Tanzania: case study of replacing chloroquine with sulfadoxine-pyrimethamine as the first line drug. Malar J 4: 51. 16242017 10.1186/1475-2875-4-51 PMC1277846

[8] Ghebreyesus T, Haile M, Witten K, Getachew A, Yohannes M, et al (2000) Household risk factors for malaria among children in the Ethiopian highlands. Trans R Soc Trop Med Hyg 94: 17–21. 10748890 10.1016/s0035-9203(00)90424-3

[9] Balls MJ, Bødker R, Thomas CJ, Kisinza W, Msangeni HA, et al (2004) Effect of topography on the risk of malaria infection in the Usambara Mountains, Tanzania. Trans R Soc Med Hyg 98(7): 400–8. 10.1016/j.trstmh.2003.11.005 15138076

[10] Oesterholt M, Bousema J, Mwerinde O, Harris C, Lushino P, et al (2006) Spatial and temporal variation in malaria transmission in a low endemicity area in northern Tanzania. Malar J 5(1): 98. 17081311 10.1186/1475-2875-5-98 PMC1635725

[11] Alemu A, Tsegaye W, Golassa L, Abebe G (2011) Urban malaria and associated risk factors in Jimma town, south-west Ethiopia. Malar J 10(173): 24. 21699741 10.1186/1475-2875-10-173 PMC3128012

[12] Wolff CG, Schroeder DG, Young MW (2001) Effect of improved housing on illness in children under 5 years old in northern Malawi: cross sectional study. BMJ 322(7296), 1209–1212. 10.1136/bmj.322.7296.1209 PMC31618 11358772

[13] Hiscox A, Khammanithong P, Kaul S, Sananikhom P, Luthi R, et al.. (2013) Risk factors for mosquito house entry in the Lao PDR. PloS ONE, 8(5), e62769. 10.1371/journal.pone.0062769 PMC3659116 23700411

[14] Coleman M, Coleman M, Mabaso MLH, Mabuza AM, Kok G, et al (2010) Household and microeconomic factors associated with malaria in Mpumalanga, South Africa. Trans R Soc Med Hyg 104(2): 143–7. 10.1016/j.trstmh.2009.07.010 19732924

[15] Yé Y, Hoshen M, Louis V, Séraphin S, Traoré I, et al (2006) Housing conditions and Plasmodium falciparum infection: protective effect of iron-sheet roofed houses. Malaria J 5(1): 8. 10.1186/1475-2875-5-8 PMC1373640 16451729

[16] Lwetoijera DW, Kiware SS, Mageni ZD, Dongus S, Harris C, et al (2013) A need for better housing to further reduce indoor malaria transmission in areas with high bed net coverage. Parasites & vectors 6(1): 1–9. 23497471 10.1186/1756-3305-6-57 PMC3599311

[17] Kirby MJ, Green C, Milligan PM, Sismanidis C, Jasseh M, et al (2008) Risk factors for house-entry by malaria vectors in a rural town and satellite villages in The Gambia. Malaria J 7(1): 2. 10.1186/1475-2875-7-2 PMC2267476 18179686

[18] Gosling RD, Gesase S, Mosha JF, Carneiro I, Hashim R, et al (2009) Protective efficacy and safety of three antimalarial regimens for intermittent preventive treatment for malaria in infants: a randomised, double-blind, placebo-controlled trial. Lancet 374(9700): 1521–1532. 19765815 10.1016/S0140-6736(09)60997-1

[19] Bousema T, Drakeley C, Gesase S, Hashim R, Magesa S, et al (2010) Identification of hot spots of malaria transmission for targeted malaria control. J Infect Dis 201(11): 1764–1774. 20415536 10.1086/652456

[20] Mboera LE, Kihonda J, Braks MA, Knols BG (1998) Influence of centers for disease control light trap position, relative to a human-baited bed net, on catches of Anopheles gambiae and Culex quinquefasciatus in Tanzania. Am J Trop Med Hyg 59: 595–596. 9790436 10.4269/ajtmh.1998.59.595

[21] Guthmann JP, Llanos-Cuentas A, Palacios A, Hall AJ (2002) Environmental factors as determinants of malaria risk. A descriptive study on the northern coast of Peru. Trop Med Int Health 7(6): 518–525. 12031074 10.1046/j.1365-3156.2002.00883.x

[22] Meyrowtisch DW, Pedersen EM, Alifrangis M, Scheike TH, Malecela MN, et al (2011) Is the current decline in malaria burden in sub-Saharan Africa due to a decrease in vector population? Malar J 10: 188. 21752273 10.1186/1475-2875-10-188 PMC3160426

[23] Gosling RD, Drakeley CJ, Mwita A, Chandramohan D (2008) Presumptive treatment of fever cases as malaria: help or hindrance for malaria control? Malar J 7: 132. 18631377 10.1186/1475-2875-7-132 PMC2488354

[24] Lindsay SW, Emerson PM, Charlwood JD (2002) Reducing malaria by mosquito-proofing houses. Trends Parasitol 18: 510–14. 12473368 10.1016/s1471-4922(02)02382-6

[25] Lindsay S, Jawara M, Paine K, Pinder M, Walraven G, et al (2003) Changes in house design reduce exposure to malaria mosquitos. Trop Med Int Health 8: 512–7. 12791056 10.1046/j.1365-3156.2003.01059.x

[26] Atieli H, Menya D, Githeko A, Scott T (2009) House design modifications reduce indoor resting malaria vector densities in rice irrigation scheme area in western Kenya. Malar J 8: 108. 19454025 10.1186/1475-2875-8-108 PMC2688520

[27] Kirby MJ, Ameh D, Bottomley C, Green C, Jawara M, et al (2009) Effect of two different house screening interventions on exposure to malaria vectors and on anaemia in children in The Gambia: a randomised controlled trial. Lancet 374(9694): 998–1009. 19732949 10.1016/S0140-6736(09)60871-0 PMC3776946

[28] Massebo F, Lindtjørn B (2013) The effect of screening doors and windows on indoor density of Anopheles arabiensis in south-west Ethiopia: a randomized trial. Malaria J 12(1): 319. 10.1186/1475-2875-12-319 PMC3847214 24028542

[29] Ogoma SB, Lweitoijera DW, Ngonyani H, Furer B, Russell TL, et al (2010) Screening mosquito house entry points as a potential method for integrated control of endophagiv filariases, arbovirus, and malaria vectors. PloS Negl Trop Dis 4: e773. 20689815 10.1371/journal.pntd.0000773 PMC2914752

[30] Sturrock HJW, Novotny JM, Kunene S, Dlamini S, Zulu Z, et al (2013) Reactive case detection for malaria elimination: real-life experience from an ongoing program in Swaziland. PLoS ONE 8(5): e63830. 23700437 10.1371/journal.pone.0063830 PMC3658965

[31] Cotter CC, Sturrock HJW, Hsiang MS, Liu J, Phillips AA, et al (2013) The changing epidemiology of malaria elimination: new strategies for new challenges. Lancet 382(9895): 7–13. 23594387 10.1016/S0140-6736(13)60310-4 PMC10583787

[32] Hsiang MS, Hwang J, Kunene S, Drakeley C, Kandula D, et al (2012) Surveillance for malaria elimination in Swaziland: a national cross-sectional study using pooled PCR and serology. PloS ONE 7: e29550. 22238621 10.1371/journal.pone.0029550 PMC3253098

[33] Bousema T, Griffin JT, Sauerwein RW, Smith DL, Churcher TS, et al (2012) Hitting hotspots: spatial targeting of malaria for control and elimination. PLoS Med 9(1): e1001165 doi:10.1371/journal.pmed.1001165 22303287 10.1371/journal.pmed.1001165 PMC3269430

